# Investigation of D_2_ Receptor–Agonist Interactions Using a Combination of Pharmacophore and Receptor Homology Modeling

**DOI:** 10.1002/cmdc.201100545

**Published:** 2012-02-07

**Authors:** Marcus Malo, Lars Brive, Kristina Luthman, Peder Svensson

**Affiliations:** [a] NeuroSearch Sweden ABArvid Wallgrens Backe 20, 413 46 Göteborg (Sweden); [b] Department of Chemistry, Medicinal Chemistry, University of Gothenburg412 96 Göteborg (Sweden); [c] Department of Biomedicine, University of GothenburgP.O. Box 440, 405 30 Göteborg (Sweden)

**Keywords:** dopamine agonists, GPCRs, pharmacophore modeling, protein structure modeling, selectivity

## Abstract

A combined modeling approach was used to identify structural factors that underlie the structure–activity relationships (SARs) of full dopamine D_2_ receptor agonists and structurally similar inactive compounds. A 3D structural model of the dopamine D_2_ receptor was constructed, with the agonist (−)-(*R*)-2-OH-NPA present in the binding site during the modeling procedure. The 3D model was evaluated and compared with our previously published D_2_ agonist pharmacophore model. The comparison revealed an inconsistency between the projected hydrogen bonding feature (Ser-TM5) in the pharmacophore model and the TM5 region in the structure model. A new refined pharmacophore model was developed, guided by the shape of the binding site in the receptor model and with less emphasis on TM5 interactions. The combination of receptor and pharmacophore modeling also identified the importance of His393^6.55^ for agonist binding. This convergent 3D pharmacophore and protein structure modeling strategy is considered to be general and can be highly useful in less well-characterized systems to explore ligand–receptor interactions. The strategy has the potential to identify weaknesses in the individual models and thereby provides an opportunity to improve the discriminating predictivity of both pharmacophore searches and structure-based virtual screens.

Supporting information for this article is available on the WWW under http://dx.doi.org/10.1002/cmdc.201100545.

## Introduction

The monoaminergic receptors, including the dopamine (DA) receptors, belong to class A or the rhodopsin-like G-protein-coupled receptor (GPCR) superfamily. The dopamine receptors are classified into five types (D_1_–D_5_) and can be categorized further into two main subfamilies: the D_1_- and D_2_-like receptors.^1^ The D_1_-like receptors (D_1_ and D_5_) activate mainly adenylate cyclase, thus leading to an increase in intracellular cAMP levels, whereas the D_2_-like receptors (D_2_, D_3_, and D_4_) either inhibit adenylate cyclase or signal through other pathways.^2^ Dopamine receptors in the central nervous system (CNS) play a major role in the initiation and control of many vital brain functions such as behavior, cognition, motor activity, learning, and reward. Selective dopamine D_2_ and mixed D_1_/D_2_ receptor agonists have been used in combination with l-DOPA in the treatment of Parkinson's disease since the early 1980s.^3^

GPCRs contain seven transmembrane helices (TM1–7). The signaling state of these receptors is associated with their active conformations. Several studies have pointed to the key role that conformational changes in TM3, TM5, and TM6 have in GPCR activation.^4^–^8^ It has been suggested that the well-conserved D(E)R^3.50^Y^1^ motif at the intracellular side of TM3 is important for receptor activation of monoaminergic GPCRs. Site-directed mutagenesis studies on the α_1b_^10^ and β_2_^7^, ^11^ adrenergic receptors, for example, have shown that an interaction between Arg^3.50^ and the Glu^6.30^ residue in TM6 restrains the movement of TM6 and stabilizes the inactive state of the receptor. This ionic lock is present in the crystal structures of inactive bovine^12^ and squid^13^ rhodopsin receptors. The conformational changes in TM6 upon activation are also supported by fluorescence studies, which indicate an increased distance between TM3 and TM6.^11^, ^14^ The ionic lock is not, however, present in the inactive states of the recently solved β_1_ and β_2_ adrenergic receptor structures (adrb1 [PDB code: 2VT4], adrb2 [2RH1], respectively) or in the adenosine A_2A_ (ad2a [PDB code: 3EML]) receptor structure.^15^–^17^ In the recently published dopamine D_3_ receptor structure (drd3, 3PBL) by Chien et al.,^18^ the ionic lock is present.

The activation process of GPCRs is catalyzed by agonists, leading to a conformational change of the receptor.^6^, ^8^, ^16^, ^19^–^23^ Agonist binding initiates an outward movement of both TM5 and TM6 at the cytoplasmic side, which in turn triggers the activation of the G protein.^21^–^23^ Site-selective fluorescence labeling studies have also shown that the magnitude of fluorescence changes upon agonist binding, which is indicative of a conformational change induced by the agonist, correlates with intrinsic activity.^8^ Common structural features for dopamine and related monoaminergic receptor agonists are a basic amino function, hydrogen bond donor/acceptor groups, and an aromatic ring system. How agonists bind to the D_2_ receptor and detailed information regarding their typical key interactions have been studied by several research groups with modeling,^24^–^30^ medicinal chemistry,^31^, ^32^ and mutation studies.^27^, ^33^–^38^

Recent findings, however, reveal that monoaminergic GPCR signaling is far more complicated than previously realized: receptor dimerization seems to play a crucial role in dopamine D_2_ receptor (drd2) signaling.^39^ In addition, the receptors can adopt several different activated conformations and can also perform non-G-protein-mediated signaling via, for example, β-arrestin pathways, resulting in a range of effects.^40^–^42^ Concepts such as functional selectivity and biased ligands have emerged and will most likely influence future pharmacological assay design^43^, ^44^ as well as drug discovery efforts directed toward these targets.^45^ However, functional selectivity of agonists should not be confused with agonist receptor-subtype selectivity, which is the focus of this study.

As mentioned above, Rasmussen et al.,^46^ together with Cherezov, Rosenbaum, and co-workers,^16^ solved the 3D structure of the human β_2_ adrenergic receptor (adrb2; PDB codes: 2R4S and 2RH1). The adrb2 structure, which most likely represents an inactive conformation of the receptor, has the inverse agonist carazolol bound.^23^ Since publication of the adrb2 crystal structures, several other GPCRs have been crystallized such as the human dopamine D_3_ receptor,^18^ the A_2A_ adenosine receptor,^47^ and the turkey adrenergic receptor β_1_,^15^ together with two crystal structures of native bovine opsin.^48^, ^49^ These opsin structures do not include ligands, but they were crystallized under conditions that govern an activated conformation of the receptor. Therefore, the structures contain some of the features often recognized as typical for an active GPCR conformation. For example, an extension of the cytoplasmic end of TM5, an outward tilt of TM6 resulting in a pairing of the cytoplasmic ends of TM5 and TM6, and conformational changes in the highly conserved D(E)R^3.50^Y and NP^7.50^xxY motifs that form a binding cavity for the G protein.^47^

We recently published a selective dopamine D_2_ agonist pharmacophore model.^28^ It contains pharmacophoric features that are present in full agonists that could indicate specific key interactions with the receptor. These features are: 1) the salt bridge between the amino function in the ligand and an aspartic acid residue in TM3, denoted Asp-TM3, 2) the hydrogen bond(s) from the phenol group(s) to serine residues in TM5 (Ser-TM5), and 3) the aromatic system (Aro), which includes a direction of the π-system for optimal face-to-edge π–π interactions with hydrophobic residues in TM6.^28^

**Figure 1:**
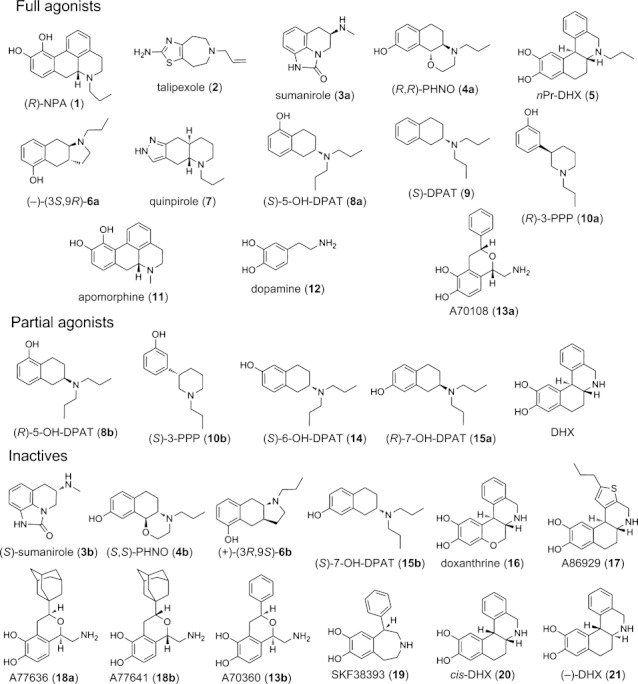
Selected D_2_ receptor full agonists **1**–**13 a**, partial agonists **8 b**, **10 b**, **14**, **15 a** and DHX, and structurally related inactives **3 b**, **4 b**, **6 b**, **12 b**, and **15 b**–**21**. For a more detailed account of the set, see reference 28.

In the present study we developed dopamine D_2_ receptor models to gain a better insight into agonist binding and the reasons behind the selectivity between full agonists and structurally similar inactive compounds. We included a more structurally diverse set of ligands (Figure 1) than those used in previously published studies. The focus in this study is characterization of the agonist binding site using a combination of 3D pharmacophore modeling and comparative (homology) modeling of the dopamine D_2_ receptor, guided by available published data. A dopamine D_2_ receptor homology model with all loops except the third intracellular loop (IC3) was built. The homology model was further compared with and modified according to our dopamine D_2_ agonist pharmacophore model.^28^

The similarities and differences between the models were analyzed in detail with regards to experimental data on agonist affinity, efficacy, and effects of binding site mutations. Deviations between the geometries of the D_2_ homology model and the pharmacophore model were used to refine the pharmacophore model with respect to the shape of the receptor binding pocket.

## Important amino acids for D_2_ agonist binding

Several interactions between the dopamine D_2_ receptor (drd2) and its ligands have been verified experimentally by mutation studies. For example, the aspartic acid residue (Asp114^3.32^) in TM3 forms a salt bridge with the basic nitrogen atom of the ligands,^26^, ^38^ and a cluster of serine residues in TM5 (Ser193^5.42^, Ser194^5.43^, and Ser197^5.46^) contribute to the binding of the catechol moiety present in many agonists.^33^, ^36^, ^37^ However, Cox et al.^36^ have shown that Ser193^5.42^ is most important for binding of catechol-containing full agonists, whereas the frequently used pyrazole-containing D_2_ receptor agonist quinpirole (**7**, Figure 1) is not as sensitive for mutations at this position. Dopamine and the partial receptor agonist DHX showed no detectable agonist activity if Ser194^5.43^ was replaced by alanine.^33^, ^36^ Wiens et al.^33^ also demonstrated that a Ser193^5.42^→Ala mutation does not affect the intrinsic activity of the full agonists (*R*)-NPA and quinpirole, for example, whereas the efficacy of DHX is drastically reduced. The binding affinity for all agonists was, however, negatively affected by the Ser193^5.42^→Ala and Ser194^5.43^→Ala mutations.^33^

TM6 contains a cluster of hydrophobic amino acids that are involved in agonist binding and in the activation of the GPCRs. In particular, Phe390^6.52^ is important for direct binding of the catechol or corresponding aromatic rings in agonists,^34^, ^50^ while Phe389^6.51^ has been suggested to interact with the positively charged basic nitrogen atom of the ligands.^50^ In binding studies, Lundström et al.^51^ have shown that a mutation of a histidine residue located in TM6 in the D_3_ receptor (His349^6.55^→Leu) affects binding of dopamine, but not binding of 7-OH-DPAT. In a D_2_ receptor mutation study performed by Gmeiner and colleagues, both (*R*)-7-OH-DPAT and dopamine were affected if His393^6.55^ was replaced with an alanine residue. Dopamine was the most sensitive of the two to this mutation.^35^ The latter study also included quinpirole binding data, and showed that the affinity of quinpirole for D_2high_ (the high-affinity state of the D_2_ receptor) was drastically decreased in the mutated receptor. However, the efficacies were unaffected.^35^ In mutation studies, the corresponding amino acid in adrb2 (Asn^6.55^) has also been shown to be important for binding of full agonists, whereas partial agonists were only moderately affected by an Asn^6.55^→Leu mutation.^52^

The second extracellular loop (EC2), which lines the binding site crevice, is also important for agonist interaction and receptor activation.^53^, ^54^ All monoaminergic GPCRs have a disulfide bridge (EC2-SS-TM3) that connects a cysteine residue in EC2 (Cys182 in drd2) with a cysteine in TM3 (Cys107^3.25^ in drd2), which thereby constrains the loop on top of the crevice. In the muscarinic acetylcholine receptor, disruption of this disulfide bond dramatically disrupts ligand binding.^55^ In addition, Noda et al.^56^ showed that removal of EC2-SS-TM3 destabilizes the high-affinity state of adrb2. Furthermore, by using the substituted-cysteine accessibility method (SCAM), Shi and Javitch have shown that Ile184 and Asn186 located in EC2 in drd2 contribute to the binding site crevice, and are therefore available for direct ligand interactions.^54^ In the dopamine D_3_ receptor (drd3) structure (3PBL), the corresponding asparagine residue (Asn185) was shown to be directed toward TM4 and not toward the binding crevice, as for drd2.^54^ Of all GPCRs, drd3 shares the highest sequence identity with drd2 (78 %)^18^ and therefore might be expected to be the most suitable template for homology modeling of drd2. However, we do not consider this to be the case in modeling D_2_ agonist–receptor interactions because the drd3 structure is crystallized with the antagonist eticlopride present in the binding pocket. Eticlopride binds closer to the extracellular side than carazolol does in adrb2 and it is unable to form a face-to-edge π–π interaction with Phe^6.52^, an interaction that is crucial for agonist binding.^34^, ^50^ In homology modeling, the template structure of conserved amino acids in the two sequences will be enforced on the model of the target protein (see below) and therefore a receptor structure with high homology but in a different conformational state can be less suitable as a template than a structure with lower sequence homology. Therefore, in the present study we chose the adrb2 structure (2RH1) as the template in the homology modeling procedure; 2RH1 has a resolution of 2.4 Å, which is the highest resolution of all crystal structures of monoaminergic GPCRs obtained so far. The main difference between the 2RH1 structure and the true receptor structure is that the third intracellular loop (IC3) had to be replaced with T4 lysozyme (T4L) in order to stabilize the receptor for crystallization. The D_2_ receptor model was built with a D_2_ receptor agonist present to induce an active-state binding site conformation. By combining the pharmacophore and receptor modeling approach (using all available SAR, mutational, and structural information) we aim at gaining a deeper understanding of the features that govern D_2_ receptor agonism.

## Results and Discussion

### Sequence alignment

An initial sequence alignment between the human adrenergic β_2_ receptor (adrb2, 2RH1^2^) and drd2 was performed using Clustal W (version 2.0.10).^57^ The program produced a correct alignment for the first five helices, but not for TM6 and TM7. The removal of IC3 from both sequences, which is considerably longer in drd2 than in adrb2, allowed a satisfactory alignment of all seven helical regions. The Clustal W alignment obtained initially (see figure 1 in the Supporting Information) was checked carefully in the non-conserved positions close to the binding site and in the loop regions.

**Figure 2:**
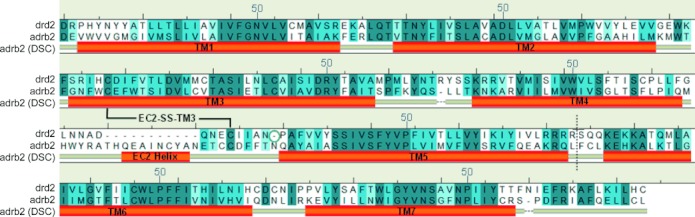
The final sequence alignment of the human adrenergic β_2_ receptor (adrb2, 2RH1) and the human dopamine D_2_ receptor (drd2). The adrb2 (DSC) bars indicate the transmembrane (TM) helix regions and the second extracellular loop helix (EC2 Helix) in the adrb2 structure. The amino acid sequence for lysozyme in adrb2 and the third intracellular loop (IC3) in drd2 between TM5 and TM6 were excised. This is marked with a dashed line. The ring at the N terminus of TM5 indicates the gap where the alanine residue was introduced to prevent Pro188^5.37^ from being forced into the second extracellular loop (EC2; see text). Amino acids marked in dark blue indicate fully conserved positions, medium blue residues have highly similar physicochemical character, and light blue residues have less similar physicochemical character. The conserved cysteine bridge between TM3 and EC2 (EC2-SS-TM3) is indicated. The most conserved residue in each helix is marked with the index 50.

**Figure 3:**
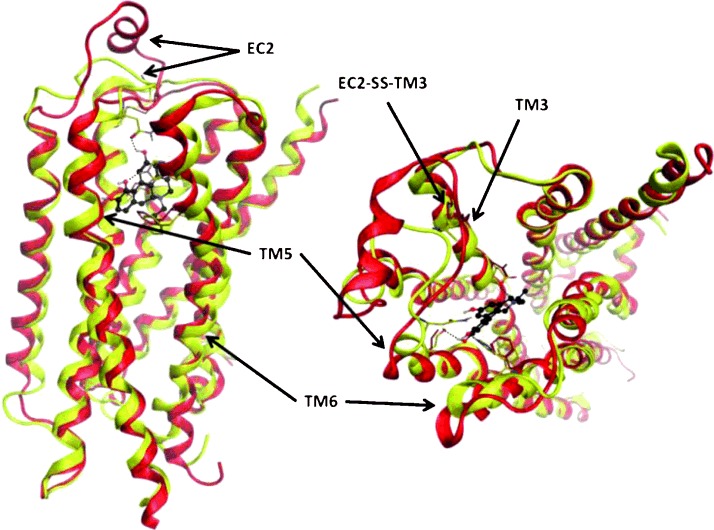
Two orthogonal views of the dopamine D_2_ receptor (drd2) homology model (yellow) with the full agonist (*R*)-2-OH-NPA present in the binding site, and the structure of the adrenergic β2 receptor (adrb2; 2RH1) in red. Some interacting amino acids of drd2 are included together with the corresponding residues in adrb2. These structures differ particularly in the second extracellular loop (EC2), but also in the upper part of transmembrane helix 5 (TM5) and TM6, where important interacting amino acids are positioned.

Manual adjustments in some parts of the sequence alignments were made with the purpose of improving the final homology model (Figure 2). The adjustments in the initial alignment were:

TM4: The sequence LLTKN in adrb2 was moved to fill a gap at the N terminus of TM4, and the sequence C^4.58^PLLF in drd2 was moved to fill a gap between Ser^4.57^ and Cys^4.58^.
EC2: The sequence DQNECIIAN in drd2 was aligned with amino acids located in the short helix present in adrb2 (EC2 helix, Figures 2 and 3). The target sequence has a considerably shorter loop than the template (11 residues shorter). QNECIIAN were moved toward the N terminus of TM4. The cysteine bridge EC2-SS-TM3 in drd2 was aligned with the corresponding cysteine bridge in the template sequence. The EC2 residues Asn186 and Ile184 in drd2 are important for ligand interactions,^54^ and were therefore aligned with the corresponding Thr and Phe residues in adrb2, which point downward to the binding crevice.
IC3: The lysozyme insert was removed from adrb2, as was the corresponding sequence in drd2.
TM7: The sequence CRS in adrb2 was moved to remove a gap at the C terminus of the helix.

The sequence similarity between adrb2 and drd2 in the manually adjusted alignment was 35 % in total, 41 % in the helix regions, and 57 % in the binding pocket. The binding pocket is defined by amino acids within 3.5 Å from the D_2_ receptor agonist (−)-(*R*)-2-OH-NPA,^32^ which was used as environment^3^ for induced fit during the homology modeling procedure.

### D_2_ homology modeling

In the applied homology modeling procedure,^4^ all heavy atoms of strictly conserved residues in the target model inherited their coordinates from the template. In the non-conserved region the backbone geometry was copied. Conserved disulfide bonds were also copied to the model. Non-aligned regions where the backbone coordinates were indefinite (i.e., regions with deletions or insertions that are often located in loops) were modeled based on fragments from high-resolution regions of proteins in the RCSB Protein Data Bank^59^ (PDB).^60^ The fragment search included a clustering algorithm based on similarity of anchoring regions (each fragment consists of two anchor regions and a central region). The fragments were anchored to the conserved and initially modeled residues, and a contact energy function was used to rank fragment candidates, taking into account all atoms from conserved residues and any specified environment atoms. The coordinates for the chosen fragment structure were then copied to the homology model. Once all the loop structures had been selected, the side chains of the non-conserved residues were constructed.

Side chains were modeled from data assembled from an extensive rotamer library generated by systematic clustering of high-resolution structures in the PDB. A deterministic procedure based on unary quadratic optimization (UQO) was then run to select an optimal packing.^61^ After all backbone segments and side chain conformations had been chosen for an intermediate model, hydrogen atoms were added to complete the valence requirements. The model was then submitted to a series of energy minimizations before the final preparation of the model was scored and written to an output database. The number of main chain models to be generated was specified in the program. In addition, structures with variations in the side chain conformations were generated for each model; however, the first side chain model was always built with the UQO procedure. The force field used for the receptor modeling in this study was Amber99^62^ with R-field solvation, as implemented in the MOE software.^58^

The cysteine bridge between Cys182 and Cys107^3.25^ (EC2-SS-TM3) in drd2 makes the N-terminal stretch of the EC2 loop more constrained than in the template structure (adrb2) due to the shorter sequence between the cysteine bridge and TM5 in drd2 (Figure 2). In an initial homology model, Pro187^5.36^ at the N terminus of TM5 was positioned in the loop (EC2), although prolines are known to introduce kinks or terminate helices. This is a consequence of the homology modeling method being so highly governed by the template structure. To prevent Pro187^5.36^ from being forced into the loop region, we used a novel strategy based on the introduction of an additional alanine residue just before the proline, which allowed proline to start the helix instead (marked with a green ring in Figure 2). The introduced alanine residue was then manually deleted, which left a gap in the structure, but a counterclockwise rotation around the center of the lower part of TM5 (after the proline kink) shortened the distance between Pro187^5.36^ and Asn186 in EC2. These two amino acids were reconnected, and a restrained energy minimization was performed in which everything was fixed except the N terminus of TM5, the EC2 with the cysteine bridge, and the C terminus of TM3. In the resulting structure, TM5 begins with Pro187^5.36^ and becomes slightly tilted inward compared with the initial homology model, which was highly similar to the template structure in that region. A structural superposition of the final model and the template is shown in Figure 3. In the final model the bulky residues Ile184 and Asn186 in EC2 are directed downward into the binding pocket, as suggested by Shi and Javitch,^54^ which may, therefore, influence ligand binding.

**Figure 4:**
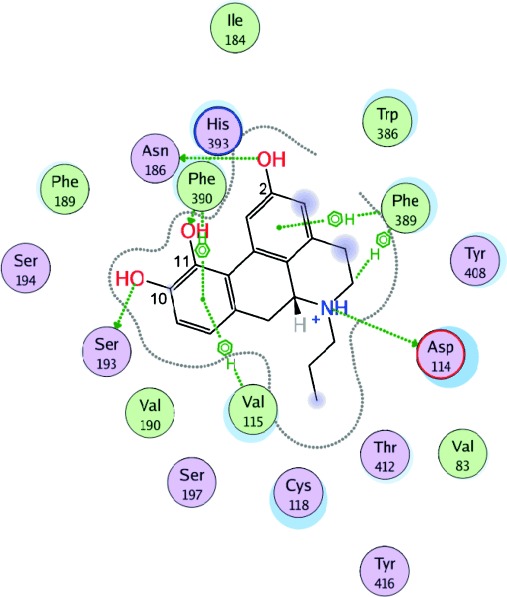
Schematic view of the interactions between the full agonist (*R*)-2-OH-NPA and the dopamine D_2_ receptor homology model. The typical catecholamine agonist–receptor key interactions with Asp114^3.32^ Ser193^5.42^ and Phe390^6.52^ are shown, together with the interactions between the hydroxy group at the 11-position in (*R*)-2-OH-NPA and His393^6.55^. In addition, the hydroxy group at the 2-position participates in a hydrogen bond with Asn186 in EC2, and Phe389^6.51^ forms a π–π interaction with the monohydroxylated phenyl group of the ligand. The characteristic propyl/allyl pocket is also indicated, located between the residues Val83^2.53^, Cys118^3.36^, Trp386^6.48^, and Tyr416^7.43^. Amino acids in purple are polar, while green residues are hydrophobic. The blue shades indicate ligand–receptor solvent accessibility.

To gather more information regarding the structure of the agonist binding site in drd2 and the key interactions in agonist binding, the D_2_ receptor agonist (−)-(*R*)-2-OH-NPA^32^ was present as environment in the binding site during the homology modeling procedure. (−)-(*R*)-2-OH-NPA is a structurally rigid full D_2_ agonist that contains not only the typical D_2_ agonist functional groups, such as a propyl-substituted basic nitrogen atom and a catechol moiety, but also a hydroxy group at the 2-position (Figure 4). This hydroxy group may interact with Asn186 in EC2 via hydrogen bonding, which would stabilize ligand binding and steer the Asn186 side chain toward the binding pocket.^54^

During the modeling procedure, 20 homology models were generated independently, and for each model all side chain conformations were sampled three times. The backbone structure of the models varied mainly in EC2 and IC2, while helical regions showed very little variation. The side chain conformations differed mainly in the two loop regions in the different homology models. Minor differences were also observed in some helical regions where there was more than one optimal packing solution. The structural quality of the 60 homology models obtained was inspected carefully. The focus was directed at the agonist binding site region and the important interacting amino acids (Figure 4). The following protein structural properties were evaluated: 1) the bond lengths, angles and dihedrals of the protein backbone; 2) Ramachandran plots of ϕ–ψ dihedrals (general, glycine, proline, and pre-proline; for explanations, see figure 2 in the Supporting Information); 3) side chain rotamer quality; and 4) non-bonded amino acid steric clashes. To refine the model, hydrogen atoms were added to the ligand, and the ionization and tautomeric states of the ligand–receptor complex were determined. The complex was refined further by energy minimization with (−)-(*R*)-2-OH-NPA present in the binding site with motion restrictions on all heavy atoms. This step was followed by an unconstrained energy minimization. The overall geometry of this model was investigated and evaluated further with the Procheck program.^63^ When excluding glycines and prolines, 199 residues (83 %) belonged to the most favored region of the Ramachandran map, 37 (16 %) in the allowed, and three (1 %) in the generously allowed region (according to Procheck). No residues belonged to disallowed regions. All main chain and side chain geometries were designated to the “better” class, except for ϕ torsions of Asn176 and Cys182 in the EC2, which were 148° and 130°, respectively. Six close contacts were listed, all between the receptor and the ligand, of which four included hydrogen atoms involved in hydrogen bonds. Close contacts are defined as pairs of non-bonded atoms within a distance of 2.6 Å from one another.

### The selected D_2_ agonist induced receptor model

**Figure 5:**
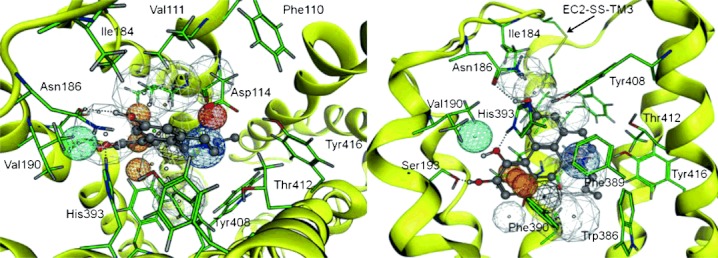
Two orthogonal views of the recently published selective dopamine D_2_ agonist pharmacophore model^28^ superimposed into the D_2_ homology model. Transmembrane helix 6 (TM6) is not shown on the side view (right), but the side chains of the interacting amino acids Phe389^6.51^, Phe390^6.52^, and Trp386^6.48^ are still included. The positioning of the anion feature (red) superimposes well with the aspartic acid Asp114^3.32^, as well as the position and direction of the aromatic system (orange), while the hydrogen bonding feature, together with the excluded volumes, mismatch with the receptor.

The final model has an RMSD of 2.1 Å in relation to the template structure for all Cα atoms and 1.5 Å for Cα atoms of the transmembrane region. The volume of the orthosteric binding pocket is 371 Å^3^. High structural similarity was observed for the backbone region except in the EC2, where the loop in the template adopts a helical structure (Figures 2 and 3). In addition, TM5 and TM6 are gently tilted inward at the extracellular side, slightly sealing the binding pocket (Figure 3). According to Mobarec et al. the TM regions of GPCRs differ more toward the extracellular side.^64^ The binding site amino acids in drd2 participate in the key interactions with the agonist (−)-(*R*)-2-OH-NPA, described in detail above. The distance from the oxygen atom in the C10 hydroxy group in 2-OH-NPA (the *para* position in its dopamine substructure) to the oxygen atom of the hydroxy group in Ser193^5.42^ is 3.0 Å, and the O-H-O(Ser193^5.42^) angle is 164° (Figure 4). The oxygen atom at the corresponding *meta* position interacts with the imidazole NH group in His393^6.55^ with a distance of 2.9 Å between the heavy atoms and an O-H-N(His393^6.55^) angle of 157°. The other nitrogen atom of the imidazole ring in His393^6.55^ interacts with the phenolic function in Tyr408^7.43^ (Figure 5). The oxygen atom in Ser197^5.46^ is 4.9 Å away from the oxygen of the *meta*-hydroxy group in 2-OH-(*R*)-NPA, and therefore does not interact directly with the ligand, but instead forms a hydrogen bond with Ser193^5.42^. This is in agreement with mutation studies^33^ performed by Wiens and co-workers in which the affinity and efficacy of (*R*)-NPA toward drd2 were only slightly affected by the Ser197^5.46^→Ala mutation. Mutation of Ser193^5.42^ or Ser194^5.43^, however, significantly decreased the receptor affinity for several agonists. The hydroxy group at the 2-position of (−)-(*R*)-2-OH-NPA interacts with the carbonyl oxygen and the NH group in the amide moiety of Asn186 located in EC2 (Figure 4 and 5).

**Figure 6:**
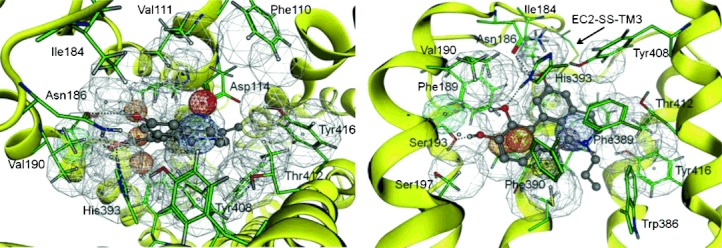
Top view of the new refined pharmacophore model based on the agonist-induced dopamine D_2_ homology model, with (*R*)-2-OH-NPA present in the binding site (left). A side view of the D_2_ homology mode with the new refined pharmacophore model is shown at right. TM6, the hydrogen atoms of the interacting amino acids, and the corresponding excluded volumes are not shown. The conformation of (*R*)-2-OH-NPA is taken from the ligand–receptor homology model complex, whereas the relative positions of the pharmacophore features are tuned to generate the best hit rate.

The distance and angles between the pairs of heavy atoms are 2.9 Å and 142° (O-H-N(Asn186)) and 2.9 Å and 143° (O-H-O=(Asn186), respectively. The basic nitrogen atom of the ligand forms a hydrogen bond stabilized salt bridge with Asp114^3.32^, the distance between the heavy atoms is 2.7 Å, and the N-H-O=(Asp114^3.32^) angle is 171°. The characteristic hydrophobic *N*-alkyl/propyl pocket^65^ in the D_2_ receptor is also present in the model and is localized between the residues Val83^2.53^, Cys118^3.36^, Trp386^6.48^, Thr412^7.39^, and Tyr416^7.43^, as shown schematically in Figure 4 and partly in Figure 5, and in Supporting Information figure 3. The side chain methyl group of Thr412^7.39^ points toward the propyl chain of (−)-(*R*)-2-OH-NPA, while its hydroxy group interacts with the backbone carbonyl of Tyr408^7.43^, which in turn may further interact and stabilize the position of the His393^6.55^ residue. Tyr416^7.35^ forms a hydrogen bond with Asp114^3.32^ and stabilizes the position of the aspartic acid, which further interacts with the basic nitrogen atom of the ligand (Figures 5 and 6). In addition, Phe390^6.52^, which has been shown to be important for agonist binding,^50^ forms a face-to-edge π–π interaction with the ligand (Figures 4, 5, and 6, and figure 3 in the Supporting Information).

McRobb et al.^66^ recently published several GPCR homology models based on the adrb2 structure (2RH1), including the D_2_ receptor. Their intention was to enrich high-throughput screening (HTS) results for receptor antagonists. Although we focus on agonist binding herein, we have compared our model with their D_2_ model to identify differences. The two models differ significantly in the orthosteric binding site. The distance between one of the oxygen atoms in Asp114^3.32^ and the oxygen in the hydroxy group of Ser193^5.42^ is 10.2 Å in the McRobb model and 9.1 Å in ours, which is in accordance with the fact that we are modeling an agonist-bound conformation of the receptor, whereas they are not. Warne et al. have shown a similar distance difference in adrenergic β_1_ receptor crystal structures.^22^ They found that the length of the binding pocket, defined as the distance between Ser^5.42^ and Asn^7.39^, is contracted by 1 Å with an agonist bound relative to when an antagonist is bound. The geometry of EC2 also varies significantly; both Ile184 and Asn186 point toward the binding crevice in our model, whereas they point toward TM4 in the McRobb model.

### Comparison of the dopamine D_2_ receptor model and the selective D_2_ agonist pharmacophore model

The recently published selective D_2_ agonist pharmacophore model^28^ was superimposed into the generated structure model of the D_2_ receptor to compare interacting amino acids with the pharmacophoric features and excluded volumes. The geometry of the feature arrangement in the pharmacophore is in good agreement with the structure model, with the exception of the Ser-TM5 feature, which did not coincide with the serine residues but was instead located more toward the extracellular side close to Val190^5.39^ (Figure 5). There were also differences in the localization of excluded volumes compared with the shape of the binding site. There could be several reasons for these types of mismatches, such as technical limitations of the modeling methodologies used or lack of experimental information when building both types of models. One reason is that the pharmacophore query tool applied has angular limitations for how hydrogen bond acceptors and donors may interact with extended pharmacophore features.^67^ The OH groups in the cathecol or phenol functions that are present in the majority of the D_2_ ligands could only hit an extended donor or acceptor feature at two distinct positions separated by 120° in the plane of the aromatic ring. This must be regarded as a limitation, as reasonably strong hydrogen bonds could most certainly be formed at directions that deviate from these optima. In the drd2 model Ser193^5.42^ is located in a position available for hydrogen bonding to the agonist, but rotation out of the ring plane of the phenol or catechol hydroxy groups is then required. This type of out-of-plane rotation is not captured in the pharmacophore development, as all active ligands with phenolic hydroxy groups prefer an in-plane conformation in the absence of the binding site environment. One published example of such an out-of-plane hydrogen bond interaction is shown in the crystal structure of adrenaline, in which the *meta*-hydroxy group in the catechol moiety is rotated 71° relative to the aromatic ring to form a hydrogen bond with a structural water molecule.^68^ A second reason for the mismatch between the models in the TM5 region may be that water-mediated hydrogen bonds between the serine residues and the D_2_ ligands in the receptor model are not considered. A third reason could be that the dynamic aspect of a functional receptor is not modeled by the homology model but may be represented in the set of active and inactive ligands used in the pharmacophore modeling (i.e., there could be a better match with functionally important alternative receptor conformations different from the snapshot conformation found in the crystal). Other reasons could be possible errors introduced by structural differences between the template and target structures not picked up by the homology modeling procedure, and structural artifacts in the template caused by protein engineering or crystal packing.

On the other hand, it has been shown in several studies that the serines in TM5 are of varied importance for agonist binding in the D_2_ receptor.^33^, ^36^ For example, Payne et al.^31^ showed that the non-hydroxylated dipropylaminotetralin derivative (*S*)-DPAT is a full D_2_ receptor agonist, but with low affinity. The same study included both enantiomeric forms of a number of DPAT analogues in which hydroxy groups were differently positioned, resulting in varied affinities and efficacies. These derivatives can thus provide a deeper understanding of the involvement of the serine residues in agonist binding to the D_2_ receptor. A similar set of agonist ligands was used by Sahlholm et al.^69^ who used voltage-sensitivity measurements to investigate the importance of hydrogen bonding interactions to the serines. It was proposed that the hydrogen bonding to the serines was important for flexible ligands (e.g., *meta*-tyramine) to allow a proper interaction between the aromatic ring of the ligand and the aromatic cluster in TM6. In the DPAT series, however, Sahlholm et al. suggested that the rigidity of the ring system results in a tight interaction of the aromatic ring without the need for additional hydrogen bonding interactions. The hydrogen bonds to the serines are not considered to be crucial for agonism but that they enhance the affinity. The authors also suggest that the more constrained binding modes of the full DPAT agonists and dopamine make them voltage sensitive due to a voltage-induced rotation of TM6 upon receptor activation.^69^

### Refinement of the D_2_ pharmacophore model

As the positioning of the Ser-TM5 feature in the pharmacophore model differed significantly relative to the drd2 homology model (Figure 5), we decided to move this feature to a position more consistent with the Ser193^5.42^ position. The new Ser-TM5 feature was positioned deeper in the binding crevice based on projected hydrogen bond annotation points generated from the pharmacophore hits of low-energy conformers of the full D_2_ receptor agonists. One argument for why TM5 hydrogen bond interactions may not be crucial for D_2_ receptor agonism is that the full agonist (*S*)-DPAT has no aromatic substituents or heteroatoms in that region. The hydrogen bond interactions to the serines in TM5 may, however, contribute to agonist affinity, so the Ser-TM5 feature was therefore defined as optional in the new pharmacophore. The other features in the pharmacophore model were kept as essential, which means that they must be matched. In addition, the positioning of the excluded volumes was arranged based on the shape of the agonist binding pocket. For example, excluded volumes were introduced to cover hydrogen atoms in amino acids located within 3 Å of (−)-(*R*)-2-OH-NPA, including those involved in hydrogen bonding with the ligand. The initial radii of the excluded volumes were selected from the van der Waals radii (vdWr) proposed by Bondi,^70^ (i.e., 1.2 Å for aliphatic and 1.0 Å for aromatic hydrogen atoms). The vdWr for hydroxy and amine hydrogen atoms are not defined by Bondi, but were set at the same radii as hydrogen atoms in benzene. The sizes of the excluded volumes were tuned manually until the pharmacophore model was sufficiently selective between actives and inactives. The final radii were 2.0 for aliphatic and 1.8 Å for aromatic hydrogens. We also introduced excluded volumes (radii 2.5 Å) positioned at the center of mass of the aromatic rings to avoid clashes between perpendicularly positioned aromatic ring systems in the receptor and the ligand (Figure 6).

The alignment of the feature part of the pharmacophore model in relation to the set of new excluded volumes derived from the receptor model was tuned manually and evaluated by the hit rate of the ligand training set. The alignment that resulted in the best discrimination between actives and inactives was selected. The new refined pharmacophore model was screened against two conformational ensembles of D_2_ ligands (structures shown in Figure 1; for a full account of the set, see reference 28) generated with both MOE^58^ (mmffs)^71^ and MacroModel^72^ (OPLS)^73^ software and Born solvation (water). The results of the initial screen were good and compared well with those obtained using the previously published pharmacophore model,^28^ despite the fact that the hit criterion for the Ser-TM5 feature was changed from essential to optional (Table 1). The excluded oxygen feature (exclO) was kept from the old D_2_ agonist pharmacophore model to prevent doxanthrine, the inactive DHX analogue, from fitting into the model. Unlike DHX, doxanthrine has an ether function that is directed toward a hydrophobic environment in the receptor. To gain a deeper understanding of the properties that govern agonist affinity and efficacy, we investigated in detail how the different ligands that fit the new pharmacophore model interact with the receptor binding site. The best hit of each compound in the pharmacophore model was evaluated by measuring the hydrogen bond distances and angles between the ligand and the amino acids Ser193^5.42^ and His393^6.55^ (Table 1).^33^, ^35^, ^36^ The optimal distance for hydrogen bonding is ∼2.8 Å between the heavy atoms, but because the receptor is flexible we consider distances between 2.4 and 3.8 Å to be acceptable. The angle between the heavy atom and the hydrogen of the ligand to oxygen in the interacting amino acid (N/O-H-O(Ser193^5.42^) and N/O-H-N(His393^6.55^)) should ideally be 180±40° (Table 1).

**Table 1:** Comparison of the search results from the old and new D_2_ agonist pharmacophore models for two different ensembles of generated conformations.

		New pharmacophore model								Old pharmacophore model					
Ligand		MOE stochastic search Born solvation MMFF94(S)[a]				MacroModel serial torsion search GB/SA solvation OPLS2005[b]				MOE stochastic search Born solvation MMFF94(S)[a]			MacroModel serial torsion search GB/SA solvation OPLS2005[b]		
		Δ*E*[c]	RMSD[d]	#c/#h[e]	Δ*E*[c]	RMSD[d]	S193 *d* [Å](∢ [°])[f]	H393 *d* [Å](∢ [°])[g]	#c/#h[e]	Δ*E*[c]	RMSD[d]	#c/#h[e]	Δ*E*[c]	RMSD[d]	#c/#h[e]
(*R*)-NPA (**1**)[h]	Full	0.0	0.26	12/3	0.6	0.22	***m*****3.7(129)** *p*2.4(163)	3.6(157)	21/11	0.0	0.59	12/6	0.0	0.45	21/19
Talipexole (**2**)[h]	Full	0.0	0.43	44/28	0.0	0.43	2.4(171)	**4.1(151)**	28/15	0.0	0.54	44/22	0.0	0.51	28/14
Sumanirole (**3 a**)	Full	0.0	0.26	5/1	0.3	0.32			3/1	0.0	0.32	5/2	0.0	0.23	3/2
(*R*,*R*)-PHNO (**4 a**)	Full	0.0	0.3	6/1	1.3	0.30	2.7(144)	**4.6(152)**	12/6	0.0	0.51	6/4	0.0	0.50	12/12
*n*Pr-DHX (**5**)[h]	Full	0.4	0.39	3/1	0.0	0.36	*m*3.0(147) ***p*****2.9(139)**	**4.5(144)**	57/6	0.4	0.47	3/1	0.0	0.44	57/24
(−)-(3*S*,9*R*)-**6 a**[h]	Full	0.0	0.25	5/3	0.0	0.25	**3.7(128)**	3.6(158)	12/6	0.0	0.53	5/5	0.0	0.54	12/12
Quinpirole (**7**)[h]	Full	0.1	0.26	5/1	1,1	0.21		**4.4(150)**	9/4	0.0	0.38	5/5	0.0	0.36	9/7
(*S*)-5-OH-DPAT (**8 a**)	Full	1.7	0.26	78/12	2.2	0.26	**3.7(128)**	3.6(164)	140/11	1.7	0.57	78/21	2.1	0.57	140/30
(*S*)-DPAT (**9**)	Full	1.7	0.27	79/12	2.2	0.27			79/6						
(*R*)-3-PPP (**10 a**)[h]	Full			26/0					43/0	3.5	0.68	26/3			43/0
Apomorphine (**11**)[h]	Full	0.0	0.19	2/1	0.0	0.21	2.4(164)	3.6(157)	4/4	0.0	0.56	2/1	0.0	0.47	4/4
Dopamine (**12**)	Full	0.0	0.57	8/4	0.0	0.58	***m*****3.8(137)** *p*2.6(145)	*m*3.8(167)	20/12	0.0	0.66	8/3	0.0	0.61	20/6
A70108 (**13 a**)	Full			6/0					10/0	2.2	0.57	6/2	2.8	0.58	10/1
(*R*)-5-OH-DPAT (**8 b**)	Partial	0.0	0.67	75/13	2.3	0.66	**3.5(132)**	3.7(158)	140/14			75/0			140/0
(*S*)-3-PPP (**10 b**)[h]	Partial			18/0					54/0			18/0			54/0
(*S*)-6-OH-DPAT (**14**)	Partial	1.7	0.26	82/14	2.2	0.27	**4.0(179)**		154/14	1.7	0.53	82/22	2.1	0.54	154/34
(*R*)-7-OH-DPAT (**15**)	Partial	0.0	0.67	84/13	2.2	0.27	2.6(148)		152/16	1.8	0.65	84/16	2.1	0.63	152/28
DHX	Partial				0.0	0.32	*m*2.9(150) *p*2.8(143)								
(*S*)-Sumanirole (**3 b**)	Inactive			5/0					3/0			5/0	1.5	0.62	3/1
(*S*,*S*)-PHNO (**4 b**)[h]	Inactive			6/0					16/0			6/0			16/0
(+)-(3*R*,9*S*)-**6 b**[h]	Inactive			5/0					12/0			5/0			12/0
(*S*)-7-OH-DPAT (**15 b**)	Inactive	1.7	0.67	79/12	2.8	0.25	**3.8(128)**		151/5			79/0			151/0
Doxanthrine (**16**)	Inactive			2/0					16/0			2/0			16/0
A86929 (**17**)	Inactive			11/0					48/0			11/0	0.0	0.64	48/23
A77636 (**18 a**)	Inactive			3/0					11/0			3/0			11/0
A77641 (**18 b**)	Inactive			3/0					11/0			3/0			11/0
A70360 (**13 b**)	Inactive			5/0					15/0			5/0			15/0
SKF38393 (**19**)	Inactive			5/0					22/0			5/0			22/0
*cis*-DHX (**20**)	Inactive			6/0					12/0			6/0			12/0
(−)-DHX (**21**)	Inactive			2/0					14/0			2/0			14/0

[a] The energy cutoff for conformations generated in MOE is 4 kcal mol^−1^.

[b] The energy cutoff for conformations generated in MacroModel is 16.7 kJ mol^−1^ (∼4 kcal mol^−1^).

[c] The relative energy [kcal mol^−1^] of the conformer that fit the pharmacophore model, related to the most stable conformer in the ensemble.

[d] Root of the mean square distance between the center of the pharmacophore features and their matching ligand annotation points.

[e] #c: number of conformations generated using the assigned method; #h: number of conformations that hit the pharmacophore model.

[f] Hydrogen bond distance and angle between heavy atoms of Ser193 of the receptor and the best hits in the new pharmacophore model (O–H–O).

[g] Hydrogen bond distance and angle between heavy atoms of His393 of the receptor and the best hits in the new pharmacophore model (HA–H–N).

[f,g] The *para* and *meta* positions of the hydroxy groups in the dopamine substructure are respectively indicated by *p* and *m*; values in boldface are just outside the permitted values (2.4–3.8 Å and 180±40°).

[h] The tertiary amine is considered chiral, and two different configurations have been used in the modeling.

### Results of the pharmacophore model search

The same set of D_2_ receptor ligands (13 full agonists, five partial agonists, and 12 structurally similar inactives; Figure 1) used in the previously published pharmacophore modeling study^28^ were screened against the new pharmacophore model based on the dopamine D_2_ receptor model. All full agonists except (*R*)-3-PPP and A70108 (11/13 OPLS generated set of ligands), and all but one ((*S*)-3-PPP) of the partial agonists (4/5) fit into the pharmacophore model. In addition, the model excluded all but one of the inactives ((*S*)-7-OH-DPAT) (1/12) (Table 1). The main reason that 3-PPP did not fit the model is the perpendicular orientation between the piperidine and phenol rings in low-energy conformations. A70108, which has a single bonded phenyl ring attached to an isochromane scaffold, shows a similar orientation of the ring systems (Figure 1). All three compounds show relatively low affinity for the D_2_ receptor, and this might be explained by the conformational changes required for binding, as suggested by Liljefors and Wikstrom, for the 3-PPP enantiomers.^65^ For further evaluation we screened the pharmacophore model against a set of MMFF(s)-generated conformations, which resulted in the loss of one additional active ligand ((*R*,*R*)-PHNO) (10/13).

Some of the full and partial agonists that fit into the pharmacophore model did not fulfill the specified criteria regarding hydrogen bond distances and angles. All full agonist hits had at least one proper hydrogen bond except for (*S*)-DPAT, which lacks the hydrogen bonding substituents, and sumanirole, which may form a hydrogen bond with a distance of 4.0 Å and an angle of 128° with the Asn186 residue in the EC2 instead (see figure 4 in the Supporting Information). All hits of the partial agonists had one proper hydrogen bonding interaction except (*S*)-6-OH-DPAT, which may indicate different binding modes. The distance from the oxygen atom in the hydroxy group of (*S*)-6-OH-DPAT to the oxygen in Ser193^5.42^ is 4.0 Å (179°), which is longer than what is optimal for a hydrogen bond. Formation of a proper hydrogen bond may, however, prevent a simultaneous interaction of (*S*)-6-OH-DPAT with Phe390^6.52^, which has been shown to be important for agonist binding.^34^, ^50^ The inactive ligand (*S*)-7-OH-DPAT fit into the model, but was unable to make hydrogen bonding interactions with His393^6.55^ and Ser193^5.42^. Several of the full agonists such as (*R*)-NPA were oriented in a position in which hydrogen bonds might be formed with both Ser193^5.42^ and His393^6.55^. The partial agonist DHX interacted with His393^6.55^ at a suboptimal distance and angle, but it can form hydrogen bonds to Ser193^5.42^ with both catechol hydroxy groups. DHX lacked the efficacy for inhibition of adenylate cyclase via the Ser193→Ala^5.42^ mutated receptor, while the intrinsic activities remained for (*R*)-NPA and the non-catecholamine agonists quinpirole and (*R*)-7-OH-DPAT in the same mutated receptor.^33^

The mutation may lead to a different binding mode for DHX due to hydrogen bond formation between the *meta*-hydroxy group and His393^6.55^, causing a loss of the face-to-edge π–π interaction with Phe390^6.52^. In addition, DHX lacks the *N*-propyl substituent, which has been shown to enhance D_2_ receptor agonist binding affinity. Together, this mutation and an absence of the propyl substituent in DHX may explain the decrease in efficacy. The pyrazole-containing D_2_ receptor agonist quinpirole is completely insensitive to the Ser193→Ala^5.42^ mutation, which could be explained by the orientation of the best pharmacophore hit of quinpirole, where the pyrazole hydrogen is unable to interact with any of the hydroxy groups in the cluster of serine residues. Instead, quinpirole may form a hydrogen bond to the backbone carbonyl of Ser193^5.42^, with a distance of 3.7 Å and an angle of 152°.

**Figure 7:**
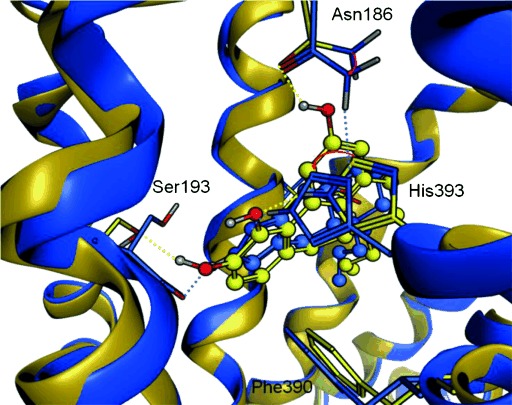
The (−)-(*R*)-2-OH-NPA (yellow ball-and-stick) generated homology model (yellow) together with the quinpirole (blue ball-and-stick) minimized receptor (blue). The rotations of the asparagine (Asn186) residue in the EC2 and the histidine (His393^6.55^) residue in TM6 are highlighted with arrows.

As mentioned above, quinpirole is sensitive to the His393^6.55^→Ala mutation, but the nitrogen atom of the pyrazole ring is not directly accessible for hydrogen bonding.^35^ Because the pyrazole is located closer to the aqueous extracellular side, there are most likely water molecules that may link the ligand and His393^6.55^ (Table 1). Another explanation for the decreased affinity in the mutant is the loss of a hydrogen bond interaction between His393^6.55^ and Asn186 in EC2. The quinpirole-induced movements of Asn186 and His393^6.55^ are shown in Figure 7 below and in figure 3 in the Supporting Information. Asn186 moves toward TM6 and interacts with His393^6.55^, which in turn moves downward to interact with the pyrazole nitrogen atom of quinpirole. Talipexole may also interact in a similar way as quinpirole and form a hydrogen bond from the amine to the backbone carbonyl oxygen of Ser193^5.42^.

To evaluate the importance of the excluded volumes for discriminating actives from inactives for the new pharmacophore model we performed a test in which we removed the volumes in the same manner as in our previous study.^28^ The new D_2_ receptor agonist pharmacophore model without excluded volumes was screened against a conformational import-generated ensemble of the D_2_ receptor ligands. The test model excluded only one partial agonist ((*S*)-3-PPP), while all other ligands fit. If the hit priority of the TM5 feature was changed to be essential, the test model succeeded in discriminating two more inactives ((*S*)-7-OH-DPAT and (*S*,*S*)-PHNO) and the full agonist (*S*)-DPAT (because it lacks the phenolic hydroxy groups). The same test was made earlier for the published D_2_ receptor pharmacophore model^28^ (with an essential TM5 feature). In that test, all of the full agonists apart from (*S*)-DPAT (12/13), two of the four partial agonists (2/4) and all but four inactives (8/12) fit into the model. The discriminating ability of the new model, which is constructed based on the shape of the binding pocket, depends more on its excluded volumes than does the old pharmacophore model.

In summary, there is a good agreement between the pharmacophore and 3D structure models. However, differences in the models that may indicate specific weaknesses in the methods and/or lack of information have been pointed out. Therefore, the combination of ligand- and structure-based modeling provides the possibility of cross-validating the models as well as a handle on how to construct more accurate models.

## Conclusions

In this study, a 3D structural model of the D_2_ receptor was developed. The previously published selective D_2_ agonist pharmacophore^28^ model, consisting of a 3D arrangement of molecular features, projected intermolecular interaction features, and excluded volumes, was aligned and compared with the receptor model. Furthermore, a new refined pharmacophore model, guided by the shape of the binding site in the receptor model, was developed. The pharmacophore and the protein structure models were constructed based on distinctly different sets of published experimental data, and are based on mutually independent assumptions and approximations. This type of combined approach helps to identify strengths and weaknesses in both strategies. The 3D model of the receptor showed good geometric quality, and the typical D_2_ receptor agonist key interactions with the receptor model were present. The selective and potent D_2_ receptor agonist (−)-(*R*)-2-OH-NPA was positioned in the binding site during the construction of the receptor model and thereby induced a conformational change of the receptor, where the binding site is shaped to accommodate an agonist. Furthermore, other important amino acids were also identified and validated based on structural information together with available binding and mutation data. We discovered, for example, that in addition to the serine residues in TM5, His393^6.55^ may also be highly important for the hydrogen bonding of agonists. This was also supported by mutation data.^35^ In addition, it was revealed from the comparison of the 3D receptor model with the published D_2_ agonist pharmacophore model, that one hydrogen bonding feature (Ser-TM5) was incorrectly positioned; therefore, a repositioning was performed to obtain a better agreement with the 3D model. The hit criterion for the Ser-TM5 feature was also redefined from being essential for hits to being optional. The hit rate of the pharmacophore search was retained, which indicates that the agonist selectivity does not depend on TM5 hydrogen bonding. However, hydrogen bonding to TM5 may still enhance the binding affinity of the agonists. Careful modeling of the loop regions was carried out, especially of the second extracellular loop (EC2), as it has been shown that the EC2 is very important for D_2_ receptor agonist binding^54^ and receptor activation.^53^

The combined pharmacophore and receptor modeling approach enabled optimal use of all relevant structure–activity, mutation, protein structure, and sequence data, and also provided a strong basis for the interpretation of the requirements for dopamine D_2_ receptor agonism based on what is known in the field to date. The combined approach also makes it easier to highlight weaknesses in each of the modeling methodologies and in the quality of the obtained models.

The new strategy can be highly useful in less well-characterized systems to explore ligand–receptor interactions and to guide the construction of each model to make it more credible for further analysis. A key ingredient to more accurate models is the diversity of the ligand set used for the construction of the pharmacophore model.

## Footnotes

1 To facilitate a comparison between different GPCRs, we use the indexing method introduced by Ballesteros and Weinstein,^9^ in which the most conserved residue in every transmembrane (TM) helix is given the index number 50. For example, the Arg residue in the highly conserved DRY motif in the cytoplasmic end of TM3 is denoted Arg^3.50^, the other residues in TM3 are then indexed relative to this position, with the previous residue as Asp^3.49^, and the subsequent as Tyr^3.51^. In addition, the absolute number of each residue in the amino acid sequence is included. In Figure 2, the index number 50 for each helix in the D_2_ receptor is highlighted.

2 One amino acid in the second extracellular loop of the β_2_ structure (2RH1) differs relative to the wild-type receptor (Asn187Glu).

3 The environment atoms (e.g., ligand structures and/or structural water molecules) are specified by the user and are included in the energy tests and minimization stages during the homology modeling procedure.

4 The modeling was performed by using tools implemented in the MOE software package.^58^

## Supporting information

Detailed facts of importance to specialist readers are published as ”Supporting Information”. Such documents are peer-reviewed, but not copy-edited or typeset. They are made available as submitted by the authors.
