# Supplementary material for: Investigation of D2 Receptor–Agonist Interactions Using a Combination of Pharmacophore and Receptor Homology Modeling
Source: ChemMedChem. 2012 Feb 7;7(3):471–82. doi: 10.1002/cmdc.201100545 (PMC3382189; doi:10.1002/cmdc.201100545)
Supplement: Supplementary file 1 [file cmdc0007-0471-SD1.pdf]

CHEMMEDCHEM
Chemistry & Drug Discovery

Supporting Information

© Copyright Wiley-VCH Verlag GmbH & Co. KGaA, 69451 Weinheim, 2012

Investigation of D_2 Receptor–Agonist Interactions Using a Combination of Pharmacophore and Receptor Homology Modeling

Marcus Malo,[b] Lars Brive,[c] Kristina Luthman,[b] and Peder Svensson*[a]

cmdc_201100545_sm_miscellaneous_information.pdf

Supporting Information

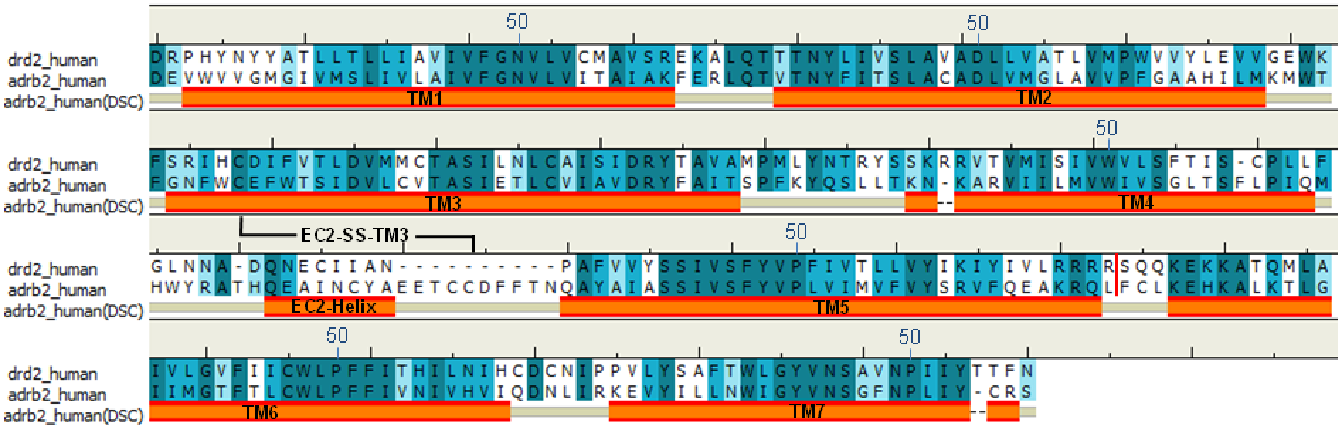

Figure 1. The initial sequence alignment of the adrenergic β_2 receptor (adrb2, 2RH1) and the dopamine D_2 receptor (drd2). The red bars indicate the transmembrane (TM) helix regions and the second extracellular loop helix (EC2-Helix) in the adrb2 structure. The amino acid sequence for lysozyme in adrb2 and the third intracellular loop (IC3) in drd2 between TM5 and TM6, were cut out. This is marked with a vertical red line. Amino acids marked in dark blue indicate fully conserved positions, medium blue residues have highly similar physicochemical character and light blue residues have less similar physicochemical character. The conserved cysteine bridge between TM3 and EC2 (EC2-SS-TM3) is indicated. The most conserved residue in each helix is marked with the index 50.

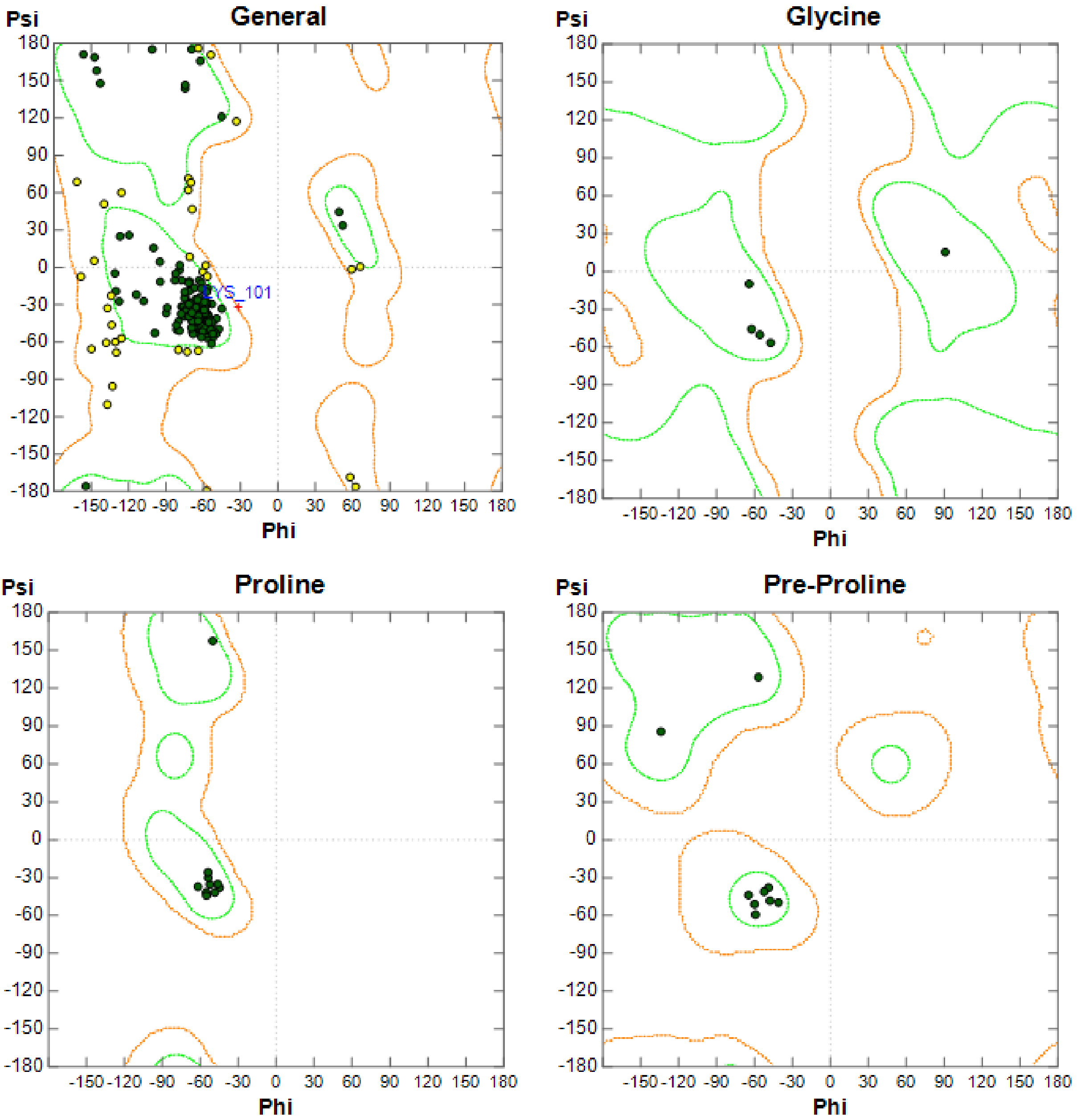

Figure 2. Ramachandran plots for glycines, prolin, pre-proline and for general residues of the selected dopamine D_2 homology model. The contours indicate allowed (orange) and core (green) regions of φ and ψ angles, and the filled green rings indicate amino acids within the core regions while the yellow rings indicate allowed regions. A red cross indicates outliers. The outlier Lys101 1st extracellular loop (EC1) and the geometry are, therefore, considered to be acceptable.

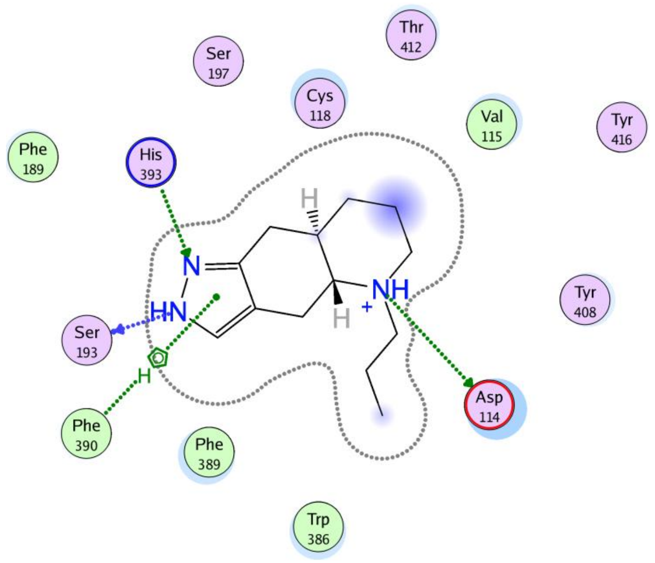

Figure 3. A schematic view of the interactions between quinpirole and the dopamine D_2 receptor homology model. The typical catecholamine agonist/receptor key interactions with Asp114$^{3.32}$ Ser193$^{5.42}$, Phe390$^{6.52}$ and His393$^{6.55}$ still remain, together with the characteristic propyl/allyl pocket. In this model the NH of the pyrazole ring in quinpirole interacts with the backbone carbonyl oxygen of Ser193$^{5.42}$. Purple-coloured amino acids are polar while the green are hydrophobic. The blue shades indicate ligand/receptor solvent accessibility.

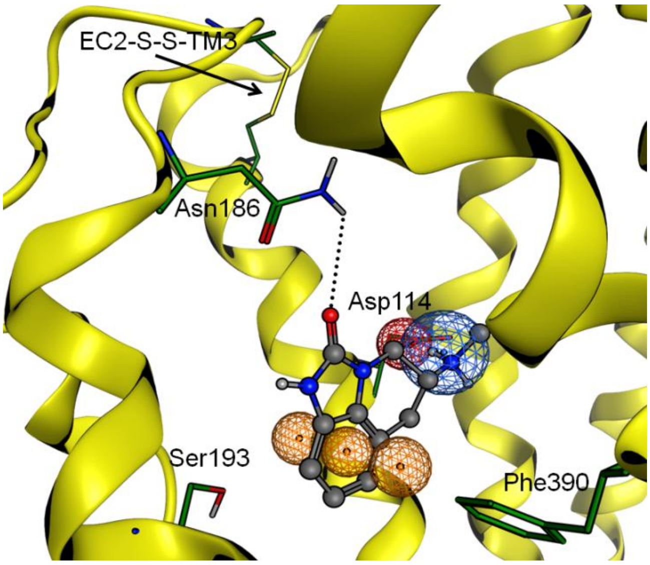

Figure 4. The pharmacophore hit of sumanirole, which is located to form a hydrogen bond with Asn186 in the second extracellular loop (EC2).
